# Fluorescence assay of dihydroorotate dehydrogenase that may become a cancer biomarker

**DOI:** 10.1038/srep40670

**Published:** 2017-01-13

**Authors:** Sheng Yin, Tsutomu Kabashima, Qinchang Zhu, Takayuki Shibata, Masaaki Kai

**Affiliations:** 1 Faculty of Pharmaceutical Sciences, Graduate School of Biomedical Sciences, Nagasaki University, 1-14 Bunkyo-Machi, Nagasaki 852-8521, Japan; 2 Department of Pharmacy, School of Medicine, Shenzhen University, 3688 Nanhai Boulevard, Shenzhen, Guangdong 518060, China

## Abstract

We developed an assay method for measuring dihydroorotate dehydrogenase (DHODH) activity in cultured HeLa cells and fibroblasts, and in stage III stomach cancer and adjacent normal tissues from the same patient. The assay comprised enzymatic reaction of DHODH with a large amount of dihydroorotic acid substrate, followed by fluorescence (FL) detection specific for orotic acid using the 4-trifluoromethyl-benzamidoxime fluorogenic reagent. The DHODH activities in the biologically complex samples were readily measured by the assay method. Our data indicate significantly higher DHODH activity in HeLa cells (340 ± 25.9 pmol/10^5^ cells/h) than in normal fibroblasts (54.1 ± 7.40 pmol/10^5^ cells/h), and in malignant tumour tissue (1.10 ± 0.19 nmol/mg total proteins/h) than in adjacent normal tissue (0.24 ± 0.11 nmol/mg total proteins/h). This is the first report that DHODH activity may be a diagnostic biomarker for cancer.

Dihydroorotate dehydrogenase (DHODH) is an enzyme in the uridine monophosphate (UMP) biosynthetic pathway that catalyses the oxidation of dihydroorotic acid (DHO) to orotic acid (Fig. 1)^1^. This enzyme mainly localises to mitochondrial membranes in mammal cells^1^. Mutation of the human DHODH gene is associated with a human genetic disorder^2^. DHODH inhibitors such as leflunomide and teriflunomide are reported to be therapeutic drugs for rheumatoid arthritis^3^,^4^,^5^ and psoriasis^6^. Several DHODH inhibitors have also been reported to have anti-malarial^7^,^8^,^9^,^10^, anti-viral^11^,^12^,^13^,^14^,^15^, and anti-tumoural^16^,^17^ effects.

An indirect colourimetric DHODH assay method was previously developed based on 2, 6-dichlorophenolindophenol (DCPIP) reduction^18^,^19^. In this reaction, DHODH catalyses DHO oxidation to orotic acid and DCPIP reduction, resulting in a colour change of DCPIP from blue to colourless that can be measured using a spectrophotometer. This method has been used to evaluate synthetic inhibitors of recombinant human DHODH^20^. However, when used for assaying DHODH activity in biologically complex samples containing mitochondrial membranes, the respiration chain complex in the mitochondrial membrane matrix significantly inhibited the redox reaction between DCPIP and DHO^21^.

We recently reported a novel fluorescence (FL) reaction with 4-trifluoromethylbenzamidoxime (4-TFMBAO) reagent for the specific quantification of orotic acid^22^. This non-FL reagent provides a strong FL signal for orotic acid without interference from other biological substances^22^. In this study, we applied this FL reaction to the assay of DHODH activity in cultured cells and in human stomach tissues. Facile, selective and sensitive FL assay of the DHODH activity was achieved by incubating DHO substrate with a small quantity of DHODH present in biologically complex samples without a need for enzyme purification.

## Results

### Description of the DHODH assay

**Figure 2:**
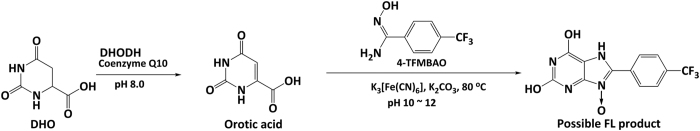
Principle of the FL assay for DHODH activity. Orotic acid is first produced from an excess of DHO substrate along with a small amount of DHODH in a sample. It is then chemically converted into a FL compound with 4-TFMBAO.

Figure 2 shows the key steps in the DHODH assay. DHO substrate was converted into orotic acid at 37 °C in the presence of K_2_CO_3_-HCl (pH 8.0), triton X-100, and coenzyme Q10. Coenzyme Q 10 is necessary to activate DHODH because it works as electron acceptor in the redox reaction^18^, and triton X-100 increases the solubility of coenzyme Q 10.

The 4-TFMBAO fluorogenic reagent selectively reacted with the orotic acid product at 80 °C for 4 min in the presence of K_3_[Fe(CN)_6_] and K_2_CO_3_ (pH 10–12), and provided a strong FL compound for orotic acid, but not for the large amount of DHO substrate or other biogenic substances such as nucleobases, nucleosides, nucleotides, amino acids, vitamins, or sugars^22^. Thus, the present assay comprises the enzymatic reaction of DHO with DHODH, followed by the FL chemical reaction of the produced orotic acid with 4-TFMBAO.

This assay format enabled the specific assay of DHODH activity in biologically complex samples such as cultured cells and tissues, and had sufficient selectivity and sensitivity for measuring the DHODH activity.

### Conditions for assay of DHODH activity in HeLa cells

**Figure 3:**
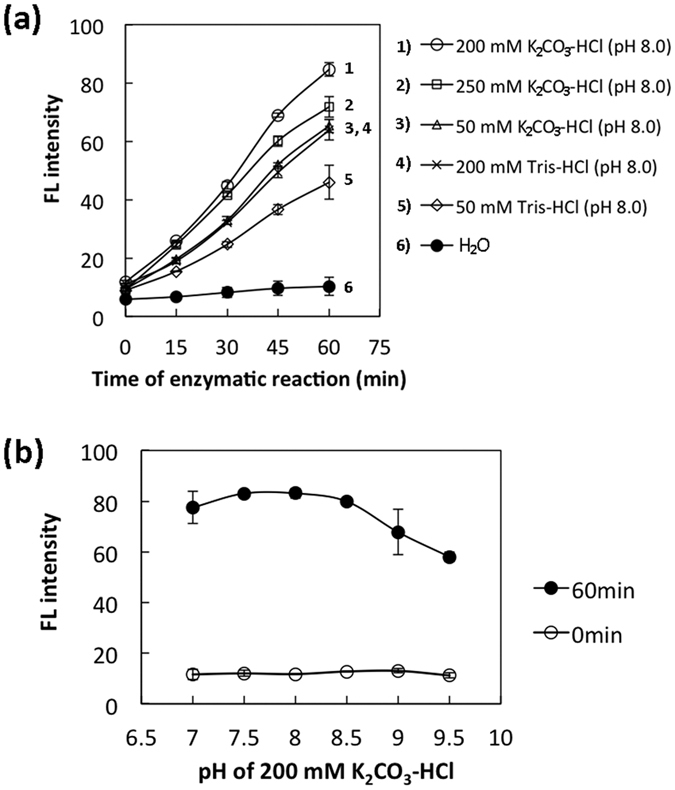
Effects of buffer concentration and pH on DHODH activity. (**a**) Effect of Tris-HCl (pH 8.0) or K_2_CO_3_-HCl (pH 8.0) concentration on DHODH activity in the presence of 500 μM DHO. (**b**) Effect of varying the pH of 200 mM K_2_CO_3_-HCl on DHODH activity. Data represent the mean ± SD of three separate experiments.

To optimise the assay conditions, cell lysate was used as the DHODH source. The effects of buffers on DHODH activity were investigated: pH 7.0–9.5, 50–250 mM K_2_CO_3_-HCl, and 50–200 mM Tris-HCl (Fig. 3). For 200 mM K_2_CO_3_-HCl (Fig. 3a), the FL intensity due to the orotic acid product was highest at a pH of between 7.0 and 8.5 (Fig. 3b). As previously reported^22^, the 4-TFMBAO reagent produced the highest FL intensity for orotic acid in the presence of 10–40 mM K_2_CO_3_ (pH ca. 11). Therefore, 200 mM K_2_CO_3_-HCl (pH 8.0) was chosen for the DHODH reaction and a final K_2_CO_3_ concentration of 40 mM (pH 11) was used for the FL reaction.

**Figure 4:**
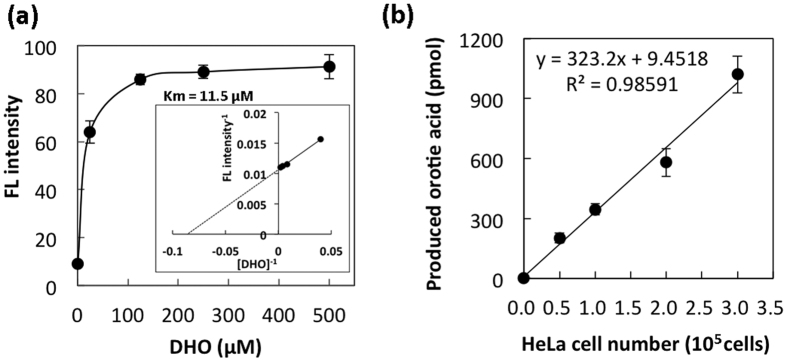
Effects of substrate concentration and HeLa cell number on DHODH activity. (**a**) Effect of varying DHO concentration on DHODH activity and *Km* value. (**b**) Relationship between DHODH activity and HeLa cell number. The enzymatic reaction was performed at 37 °C for 1.0 h. Data represent the mean ± SD of three separate experiments.

The enzymatic reaction was performed at 37 °C for 1.0 h to produce orotic acid from the DHO substrate. Figure 4a shows the effect of substrate concentration on DHODH activity and the *Km* value for the DHODH enzyme. Based on this result, a sufficient concentration (500 μM) of DHO could be selected for the present DHODH assay.

DHODH activity per 1.0 × 10^5^ cells was expressed as the amount (pmol) of orotic acid produced in 1.0 h. Cell numbers were normalised to 1.0 × 10^5^ cells in each DHODH reaction to account for differences in sample size. Usually, 2.0–3.0 × 10^5^ cells were used per assay. To measure enzyme activity, we determined the relationship between cell number and amount of orotic acid produced in a 1.0-mL reaction mixture (Fig. 4b). For the calculation of the production, the preexisting level of orotic acid at non-incubation time was subtracted from total amount of orotic acid observed at the end of the hour incubation. DHODH activity was proportional to the cell number (0.5–3.0 × 10^5^ cells). Thus, the specific activity of DHODH in the HeLa cells was 323 pmol/10^5^ cells/h.

### DHODH activity in HeLa and fibroblast cells

**Figure 5:**
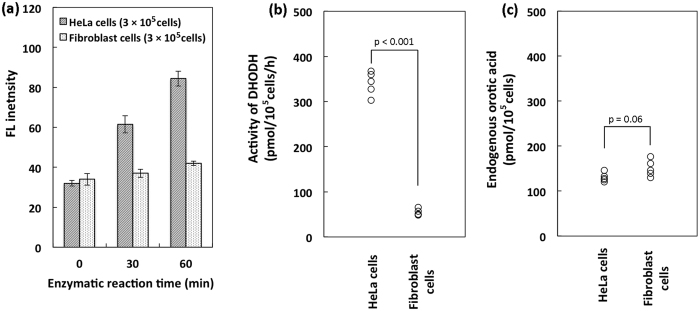
Comparison of DHODH activities and endogenous concentrations of orotic acid in HeLa cells and fibroblasts. (**a**) Increased FL intensity represents the amount of orotic acid produced by DHODH in HeLa and fibroblast cells with increasing incubation time. Data represent the mean ± SD of three separate experiments. (**b**) Specific activities of DHODH in HeLa cells and fibroblasts. (**c**) Endogenous orotic acid concentration in HeLa cells and fibroblasts. Data for b and c were obtained by five separate experiments.

FL intensity due to orotic acid increased with increasing incubation time (Fig. 5a) when either HeLa or fibroblast cells as the DHODH source were used. The specific activity of DHODH in HeLa and fibroblast cells was measured (Fig. 5b). The specific activity of DHODH was approximately 6 times higher in HeLa cells (340 ± 25.9 pmol/10^5^ cells/h) than in fibroblasts (54.1 ± 7.40 pmol/10^5^ cells/h).

We also investigated the relationship between the endogenous orotic acid content (Fig. 5c) and DHODH activity in HeLa and fibroblast cells. Endogenous orotic acid concentration was 130 ± 9.72 pmol/10^5^ for HeLa cells and 151 ± 18.6 pmol/10^5^ for fibroblasts (n = 5 each). Endogenous orotic acid levels were similar, indicating that DHODH activity is significantly different in these cell types.

### DHODH activity in malignant tumour and adjacent normal tissues

**Figure 6:**
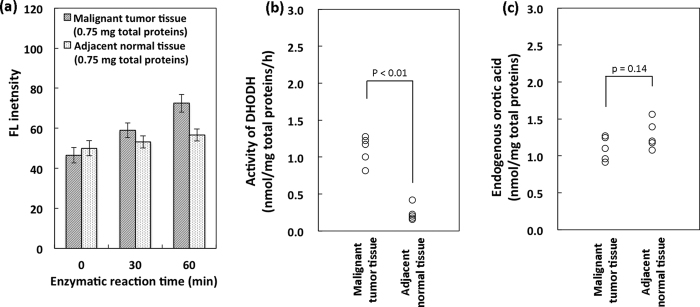
Comparison of DHODH activities and endogenous concentrations of orotic acid in stomach cancer and adjacent normal tissues. (**a**) Increased FL intensity represents the amount of orotic acid produced by DHODH in stomach cancer and adjacent normal tissues with increasing incubation time. Data represent the mean ± SD of three separate experiments. (**b**) Specific activities of DHODH in stomach cancer and adjacent normal tissues. (**c**) Endogenous orotic acid concentrations in stomach cancer and adjacent normal tissues. Data for b and c were obtained by five separate experiments.

We measured DHODH activity and endogenous orotic acid concentration in malignant stomach tumour and adjacent normal tissue from a patient with stage III cancer (Fig. 6). Tissue samples were normalised by measuring the total protein concentration to avoid variations in wet tissue weight. The total proteins in each reaction mixture were adjusted to ca. 0.75 mg.

Orotic acid was enzymatically produced in the reaction using both cancer and adjacent normal tissue samples (Fig. 6a). The FL intensity due to orotic acid increased according to the incubation time, with a similar profile to that of cultured cells. Figure 6b shows the specific activity of DHODH in malignant tumour and matched normal tissues (n = 5 each). The specific activity of DHODH in the malignant tumour tissue (1.10 ± 0.19 nmol/mg total proteins/h) was approximately 5 times higher than that in adjacent normal tissue (0.24 ± 0.11 nmol/mg total proteins/h). However, the concentration of endogenous orotic acid was similar in both malignant tumour (1.09 ± 0.16 nmol/mg total proteins) and adjacent normal (1.28 ± 0.19 nmol/mg total proteins) tissues (Fig. 6c).

### Comparison with other assay method

**Figure 7:**
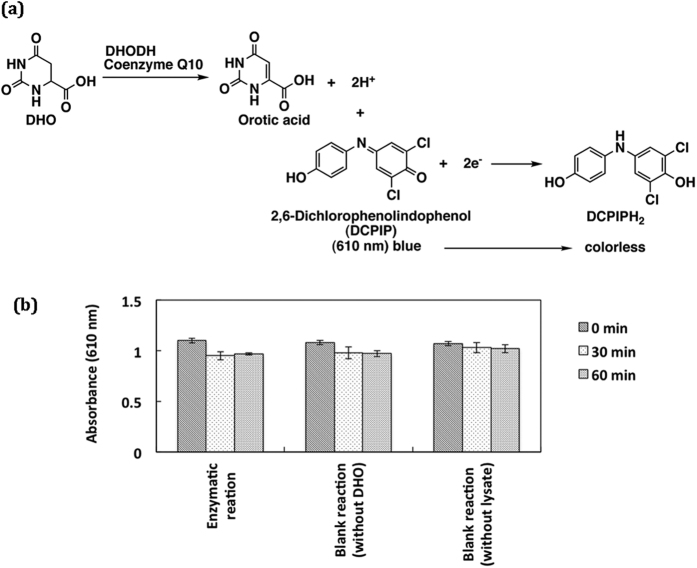
(**a**) Principle of colourimetric assay for DHODH activity. (**b**) Absorbance change of DCPIP by enzyme reaction of endogenous DHODH in HeLa cells. The enzymatic reaction was performed at 37 °C for 0, 30 or 60 min in the presence of DCPIP, DHO and cell lysate. Absorbance of DCPIC in the reaction mixture was monitored at 610 nm. Data represent the mean ± SD of three separate experiments.

Enzyme activity of recombinant DHODH has been measured for the evaluation of synthetic inhibitors of DHODH by a conventional colourimetric method^18^,^19^,^20^. Figure 7a shows the principle of the coluorimetric assay method. In the method, DHO is oxidized by the enzymatic reaction, and then reduction of DCPIP is occurred. Thus, absorbance of DCPIP in the reaction mixture should be decreased by the DHODH activity. As the results (Fig. 7b) for the assay of endogenous DHODH activity, however, DCPIP in the reaction mixture did not show the decrease of its absorbance at 610 nm depending on the incubation time. It means that DCPIP could not be reduced by the enzymatic reaction. It was thus suggested that the reductive efficiency for DCPIP was inhibited by some oxidants co-existing in the sample lysates^21^. This colourimetric assay method may require purification in advance of DHODH from the complex lysate samples. On the contrary, the present FL assay method can measure the DHODH activity in the lysates without purification of the enzyme.

## Discussion

We have developed an alternative assay method for DHODH activity in biological samples, including cultured HeLa cells and fibroblasts, and normal and malignant human stomach tissues. The assay utilised a previously reported FL reaction^22^, with 4-TFMBAO as a fluorogenic reagent. This reagent is non-FL and provided a selective, strong FL intensity for both endogenous orotic acid and enzymatically produced orotic acid under the chemical reaction conditions at 80 °C for 4.0 min in aqueous alkaline solution.

The present assay method enabled the specific activity of DHODH to be determined in the biologically complex samples. DHODH activity was apparently higher in cultured HeLa cells than in fibroblasts (Fig. 5), and also significantly higher in malignant tumour tissue than in adjacent normal tissue (Fig. 6). On the other hand, a protein, survivin has been studied for its role as a prognostic biomarker in cancer therapy^23^,^24^,^25^. Significantly higher expression of survivin was reported in gastric tumor tissues than in its adjacent normal tissues in both of its mRNA (ca. 2 folds) and protein (ca. 4 folds) levels^25^. In our research, the specific activity of DHODH in the gastric tumor (stage III) tissue was approximately 5 folds higher than that in the adjacent normal tissue. Therefore, DHODH may become a potential biomarker of cancer.

Several DHODH inhibitors have been suggested to have anti-cancer effects^16^,^17^. Our data suggest that the anti-cancer effect of DHODH inhibitors might be associated with the higher level of DHODH activity in cancer cells. Large amounts of pyrimidines are required for the rapid proliferation of cancer cells and/or malignant tissues. Thus, the pyrimidine biosynthetic pathway is activated, resulting in high DHODH expression in these cells; however, endogenous orotic acid might be rapidly metabolised to UMP^1^,^26^.

Further diagnostic studies are necessary to evaluate DHODH activity as a general cancer biomarker in other biological samples such as bloods and tissues from patients with different cancer types and different stages. The newly developed, convenient DHODH assay will technically contribute to these research approaches.

## Methods

### Chemicals

Orotic acid and coenzyme Q10 were obtained from TCI (Kyoto, Japan). 4-TFMBAO and DHO were purchased from Sigma-Aldrich (St. Louis, MO, USA). All chemicals were of analytical or guaranteed reagent grade and used without further purification. Milli-Q H_2_O was used for all reactions.

### Human tissues

Tumour and adjacent normal tissues from a patient with stage III stomach cancer were purchased from Tissue Solutions Ltd (Glasgow, UK). The supplier declared that the tissues have been obtained according to the legal and ethical requirements with the approval of an ethics committee and with anonymous consent from the donor or nearest relative. All experiments in this study were carried out in accordance with our university’s ethical guidelines.

### Cell culture

HeLa cells were cultured in Dulbecco’s modified Eagle’s medium containing 10% fetal bovine serum (FBS), 100 units/mL penicillin, 0.1 mg/mL streptomycin, and 0.25 μg/mL amphotericin B at 37 °C. Fibroblasts were cultured in minimum essential medium-alpha containing 10% FBS, 100 units/mL penicillin, 0.1 mg/mL streptomycin, and 0.25 μg/mL amphotericin B at 37 °C. Cells were harvested with trypsin at 90% confluency, washed with phosphate buffered saline (PBS), and stored at−80 °C until analysis.

### Preparation of cell and tissue lysates

Cells were counted and lysed in water (10^6^ cells/mL) at 4 °C by sonication for 10 min. Lysates were clarified at 16000 *g* for 20 min. Wet tissue was cut into tiny pieces and homogenised with a glass homogeniser (ca. 60 mg wet weight in 1.0 mL of H_2_O) for 10 min, followed by sonication for 10 min. The homogenate was clarified at 16000 *g* for 20 min. Lysates were used in the DHODH reaction and for determining endogenous orotic acid levels.

### Enzyme reaction of DHODH and FL detection of orotic acid for the assay of DHODH activity

Lysate (300 μL) was incubated in an aqueous solution (total volume, 1.0 mL) containing 500 μM DHO, 200 mM K_2_CO_3_-HCl (pH 8.0), 0.2% triton x-100, and 100 μM coenzyme Q10 at 37 °C for 0, 15, 30, 45, or 60 min. An aliquot (100 μL) of the mixture of enzyme reaction mixture or cell/tissue lysate was mixed with 100 μL of 0, 0.5, or 1.0 μM orotic acid, 50 μL of H_2_O, 250 μL of 4.0 mM 4-TFMBAO, 250 μL of 8.0 mM K_3_[Fe(CN)_6_], and 250 μL of 80 mM K_2_CO_3_ and then heated at 80 °C for 4.0 min. The reaction was stopped by cooling in an ice-water bath and the FL intensity was measured with a spectrofluorometer (FP-6300 Jasco, Tokyo, Japan): excitation and emission wavelengths were 340 nm and 460 nm, respectively.

### Conventional colourimetric assay

Lysate (300 μL) of HeLa cells was incubated in an aqueous solution (total volume, 1.0 mL) containing 500 μM DHO, 500 μM DCPIP, 200 mM K_2_CO_3_-HCl buffer (pH 8.0), 0.2% triton x-100, and 100 μM coenzyme Q10^19^. The reaction was initiated by the addition of the lysate sample. Absorbance of DCPIP in the reaction mixture was monitored at 610 nm at a periodical incubation time of 0, 30 or 60 min.

### Normalisation of sample size

To enable comparison between samples, DHODH activity and endogenous orotic acid concentration in HeLa cell and fibroblast samples were normalised to cell number (1.0 × 10^5^). DHODH activity and endogenous orotic acid concentration in tissue samples were normalised to the total protein concentration. Total protein concentration in tissue sample was measured using a colourimetric kit (Quick Start™ Bradford 1× Dye; Bio-Rad Laboratories, Hercules, CA, USA).

### Data analysis

Statistical analysis was performed using two-tailed Student’s *t* test. *P* < 0.05 was considered to be significant.

## Additional Information

**How to cite this article:** Yin, S. *et al*. Fluorescence assay of dihydroorotate dehydrogenase that may become a cancer biomarker. *Sci. Rep.* **7**, 40670; doi: 10.1038/srep40670 (2017).

**Publisher's note:** Springer Nature remains neutral with regard to jurisdictional claims in published maps and institutional affiliations.

## Figures and Tables

**Figure 1:**
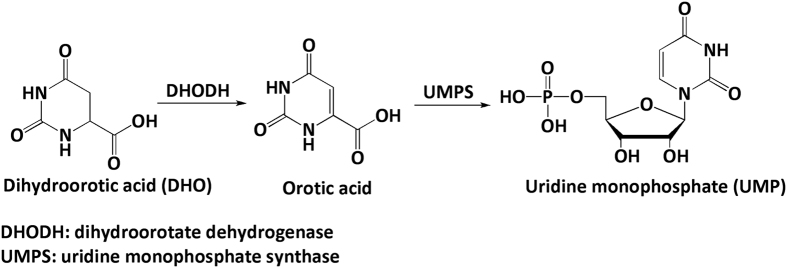
Catalytic reaction of DHODH in the UMP biosynthetic pathway. DHODH catalyses the production of orotic acid from DHO, and orotic acid is then converted to UMP in mammalian cells.
